# Why Does Not the Leaf Weight-Area Allometry of Bamboos Follow the 3/2-Power Law?

**DOI:** 10.3389/fpls.2018.00583

**Published:** 2018-05-04

**Authors:** Shuyan Lin, Lijuan Shao, Cang Hui, Yu Song, Gadi V. P. Reddy, Johan Gielis, Fang Li, Yulong Ding, Qiang Wei, Peijian Shi

**Affiliations:** ^1^Co-Innovation Centre for Sustainable Forestry in Southern China, Bamboo Research Institute, Nanjing Forestry University, Nanjing, China; ^2^Department of Mathematical Sciences, Centre for Invasion Biology, African Institute for Mathematical Sciences, Stellenbosch University, Matieland, South Africa; ^3^Center for Integrative Conservation, Xishuangbanna Tropical Botanical Garden, Chinese Academy of Sciences, Mengla, China; ^4^Western Triangle Agricultural Research Centre, Montana State University, Conrad, MT, United States; ^5^Department of Biosciences Engineering, University of Antwerp, Antwerp, Belgium

**Keywords:** allometry, Gielis equation, natural logarithm, proportionality, reduced major axis

## Abstract

The principle of similarity (Thompson, 1917) states that the weight of an organism follows the 3/2-power law of its surface area and is proportional to its volume on the condition that the density is constant. However, the allometric relationship between leaf weight and leaf area has been reported to greatly deviate from the 3/2-power law, with the irregularity of leaf density largely ignored for explaining this deviation. Here, we choose 11 bamboo species to explore the allometric relationships among leaf area (*A*), density (ρ), length (*L*), thickness (*T*), and weight (*W*). Because the edge of a bamboo leaf follows a simplified two-parameter Gielis equation, we could show that *A* ∝ *L*^2^ and that *A* ∝ *T*^2^. This then allowed us to derive the density-thickness allometry ρ ∝ *T*^*b*^ and the weight-area allometry *W* ∝ *A*^(b+3)/2^ ≈ *A*^9/8^, where *b* approximates −3/4. Leaf density is strikingly negatively associated with leaf thickness, and it is this inverse relationship that results in the weight-area allometry to deviate from the 3/2-power law. In conclusion, although plants are prone to invest less dry mass and thus produce thinner leaves when the leaf area is sufficient for photosynthesis, such leaf thinning needs to be accompanied with elevated density to ensure structural stability. The findings provide the insights on the evolutionary clue about the biomass investment and output of photosynthetic organs of plants. Because of the importance of leaves, plants could have enhanced the ratio of dry material per unit area of leaf in order to increase the efficiency of photosynthesis, relative the other parts of plants. Although the conclusion is drawn only based on 11 bamboo species, it should also be applicable to the other plants, especially considering previous works on the exponent of the weight-area relationship being less than 3/2 in plants.

## Introduction

The leaf of a plant is an important organ for transpiration, photosynthesis and heat balance (Parkhurst and Loucks, 1972; Goudriaan and Laar, 1994; Wright et al., 2004a,b). Leaf traits such as leaf area, length, width, weight, thickness, density, shape and leaf mass per area (LMA) are thus important functional indices of the leaf economic spectrum (Wright et al., 2004b; Poorter and Rozendaal, 2008). Due to their simplicity, these features have been extensively used by physiologists and ecologists to explain plant growth, developmental rate, water and nutrient use, and plant distributions (Cornelissen et al., 2003; Wright et al., 2004a,b, 2005; Shipley et al., 2006), such as for fruit development (Montero et al., 2000; Iii and Martinson, 2003; Demirsoy et al., 2004, 2005; Fallovo et al., 2008; Buttaro et al., 2015) and crop yield (Stoppani et al., 2003; Salerno et al., 2005; Peksen, 2007). These leaf functional traits are tightly related with each other. For instance, the leaf area, an important canopy parameter that directly affects light interception, light penetration, leaf energy balance and the distribution of solar radiation (Chiariello, 1984; Niinemets and Kull, 1994), can be accurately estimated by a simple equation of leaf dimensions (leaf width and length) (Gamiely et al., 1991; Whitworth et al., 1992; Uzun and Çelik, 1999), especially in crops such as rabbiteye blueberries, squash, strawberry and grapes (NeSmith, 1991, 1992; Montero et al., 2000; Demirsoy et al., 2005). The LMA, which is strongly correlated with many leaf functional, biochemical and structural traits (Poorter et al., 2009; Villar et al., 2013; Puglielli et al., 2015a,b; Millamoreno et al., 2016; John et al., 2017), tends to be small for fast-growing species (Wright et al., 2004a; Griffith et al., 2016) and is tightly related to gross leaf structure (Cornelissen and Thompson, 1997; Cornwell et al., 2008; Onoda et al., 2011).

Leaf dry weight (*W*) and leaf surface area (*A*) are two important traits for the vast majority of vascular plants (Roderick et al., 1999; Sack et al., 2003; Calvo-Alvarado et al., 2008; Pan et al., 2013). The relationship between *A* and *W* follows a power law, *W* = α *A*^β^, where α is the normalization constant and β the scaling exponent (Niklas et al., 2007; Niklas and Christianson, 2011), suggesting that the light-capturing surface per unit investment of dry mass is an important factor to leaf size.

Bamboos, the subfamily Bambusoideae of the grass family Poaceae (or Graminaceae), have been widely used for food, handicraft and construction materials. It includes 75 genera and 1,300 species, covering 25 million hectares worldwide (Liese and Köhl, 2015), with more than 500 species distributed in China. Besides the apparent differences in leaf size among bamboo species, the bamboo leaf appears to be very similar in shape across species, and its bilateral symmetry can be accurately described by a simplified Gielis equation (Lin et al., 2016). To date, the relationship between leaf area and leaf weight of bamboos have not been systematically examined. The principle of similarity (Thompson, 1917) states that the weight of an organism should follow a 3/2-power law with its surface area or proportional to its volume if the density does not change. However, the leaf weight-area allometry has been reported to greatly deviate from the 3/2-power law, with the reason yet to be clarified. We argue that this could be due to the currently unspecified scaling relationship between leaf thickness and density. Here, using data measured for 11 bamboo species, we first explore the relationship between the leaf thickness and leaf density and then derive the allometric relationship between leaf area and leaf weight, through which providing an overall explanation for the allometric relationships among leaf area, density, length, thickness and weight.

## Materials and methods

### Sample collection

We measured leaf surface area and leaf biomass values of at least 100 leaves for each of the 11 bamboo species located in the Nanjing Forestry University campus used in this study (Table 1). Leaf biomass was measured as fresh weight using ME204 (METTLER TOLEDO Equipment Limited Company, Shanghai, China; *d* = 0.0001 g) for species 1–7 in 2016, and an electronic scale with precision 0.01 g (JM-A3002; Chaozeheng Equipment Limited Company, Zhuji, Zhejiang, China) for species 8–11 in 2014. The leaves were weighed as soon as they were picked from the plants, so the loss of water in leaves can be neglected. Species 8–11 belong to *Indocalamus*, and their leaf sizes are all large. Thus, we used a low-precision electronic scale that can directly weigh large leaves.

**Table 1:** Bamboo species, sampling time and sample size of leaves.

**Code**	**Latin name**	**Sampling time**	**Number of leaves sampled**
1	*Bambusa multiplex* (Loureiro) Raeuschel ex Schultes and J. H. Schultes	Early June, 2016	200
2	*Phyllostachys edulis* (Carrière) J. Houzeau de Lehaie	Early May, 2016	200
3	*Pleioblastus argenteostriatus* (Regel) Nakai	Late April, 2016	200
4	*Pleioblastus chino* (Franchet and Savatier) Makino	Late April, 2016	200
5	*Pleioblastus kongosanensis* f. *aureostriatus* Muroi and Y. Tanaka	Mid-September, 2016	200
6	*Pseudosasa amabilis* var. *convexa* Z. P. Wang and G. H. Ye	Mid-September, 2016	200
7	*Phyllostachys incarnata* T. H. Wen	Early December, 2016	210
8	*Indocalamus pedalis* (Keng) P. C. Keng	Early July, 2014	112
9	*Indocalamus pumilus* Q. H. Dai and C. F. Keng	Early July, 2014	108
10	*Indocalamus barbatus* McClure	Early July, 2014	113
11	*Indocalamus victorialis* P. C. Keng	Early July, 2014	121

### Calculations and statistics

To connect these leaf functional traits, let *W* denote leaf weight, *V* leaf volume, ρ leaf density, *A* leaf surface area, *L* leaf length and *T* leaf (mean) thickness. Leaf shapes of bamboos have been demonstrated to follow a simplified Gielis equation (Gielis, 2003; Shi et al., 2015; Lin et al., 2016):

(1) $$\begin{array}{r} r=\frac{l}{{\left(\left|\cos\frac{\varphi }{4}|+|\sin\frac{\varphi }{4}\right|\right)}^{1/n}}. \end{array}$$

**Figure 1:**
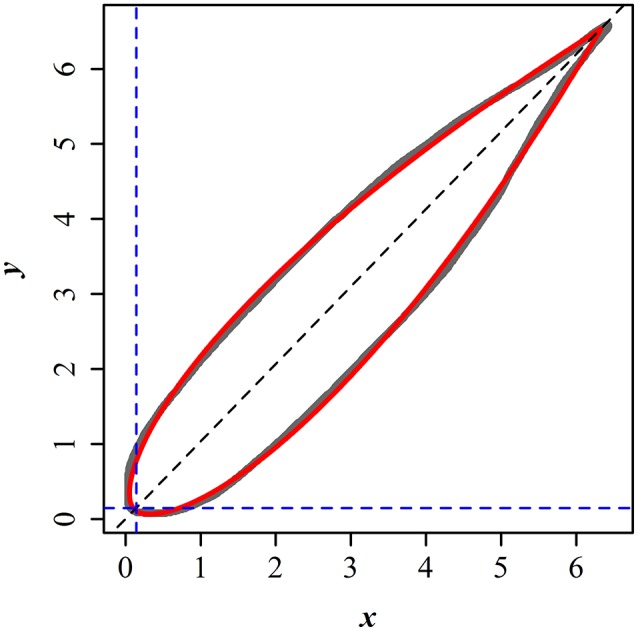
Comparison between the scanned and predicted leaf edges of *P. incarnata*. The gray curve represents the scanned leaf edge, and the red solid curve represents the predicted leaf edge by the simplified Gielis equation.

Here *r* and φ represent the polar radius and angle from the horizontal axis (i.e., polar coordinates), *l* and *n* are constants. Figure 1 illustrates the scanned edge of a bamboo leaf and the prediction from the fitted Gielis equation. Leaf length (*L*) can be described by the following equation (Shi et al., 2015):

(2) $$\begin{array}{r} L=\left(1+2^{-\frac{1}{n}}\right)\cdot l. \end{array}$$

Assuming that parameter *n* is a constant for different individuals of a same species, we could estimate the leaf surface area (*A*) as:

(3) $$\begin{array}{r} A=\frac{1}{2}\int_{2\pi }^{0}{\left[r\left(\varphi \right)\right]}^{2}d\varphi =f\left(l\right)\propto l^{2}\propto L^{2} \end{array}$$

(see Appendix for mathematical proofs).

By definition, we have

(4) $$\begin{array}{r} \rho =W/V=W/\left(AT\right) \end{array}$$

According to the principle of similarity (Thompson, 1917), we have

(5) $$\begin{array}{r} T\propto A^{1/2}\Leftrightarrow A\propto T^{2}. \end{array}$$

The above equation will be tested using the experimental data (see below for details). Let us assume that leaf density and thickness follow the following relationship:

(6) $$\begin{array}{r} \rho =\alpha T^{\beta }\Leftrightarrow \ln\;\left(\rho \right)=\ln\;\left(\alpha \right)+\beta \ln\;\left(T\right). \end{array}$$

Alternatively, this can be described as:

(7) $$\begin{array}{r} \ln\;\left(WA^{-3/2}\right)=a+b\ln\;\left(A^{1/2}\right). \end{array}$$

Here, α, β, *a*, and *b* are constants. For convenience, we can rewrite Equation (7) to

(8) $$\begin{array}{r} \ln\;\left(p_{\rho }\right)=a+b\ln\;\left(p_{T}\right), \end{array}$$

where *p* represents the proportionality. It is apparent that the slope *b* can determine the scaling relationship between leaf weight and leaf area.

(9) $$\begin{array}{r} \rho \propto WA^{-3/2}\propto A^{b/2}\Leftrightarrow W\propto A^{\left(b+3\right)/2}. \end{array}$$

If leaf density was not related to leaf thickness, *b* = 0, we would expect to have *W* ∝ *A*^3/2^, directly following the principle of similarity (Thompson, 1917).

**Figure 2:**
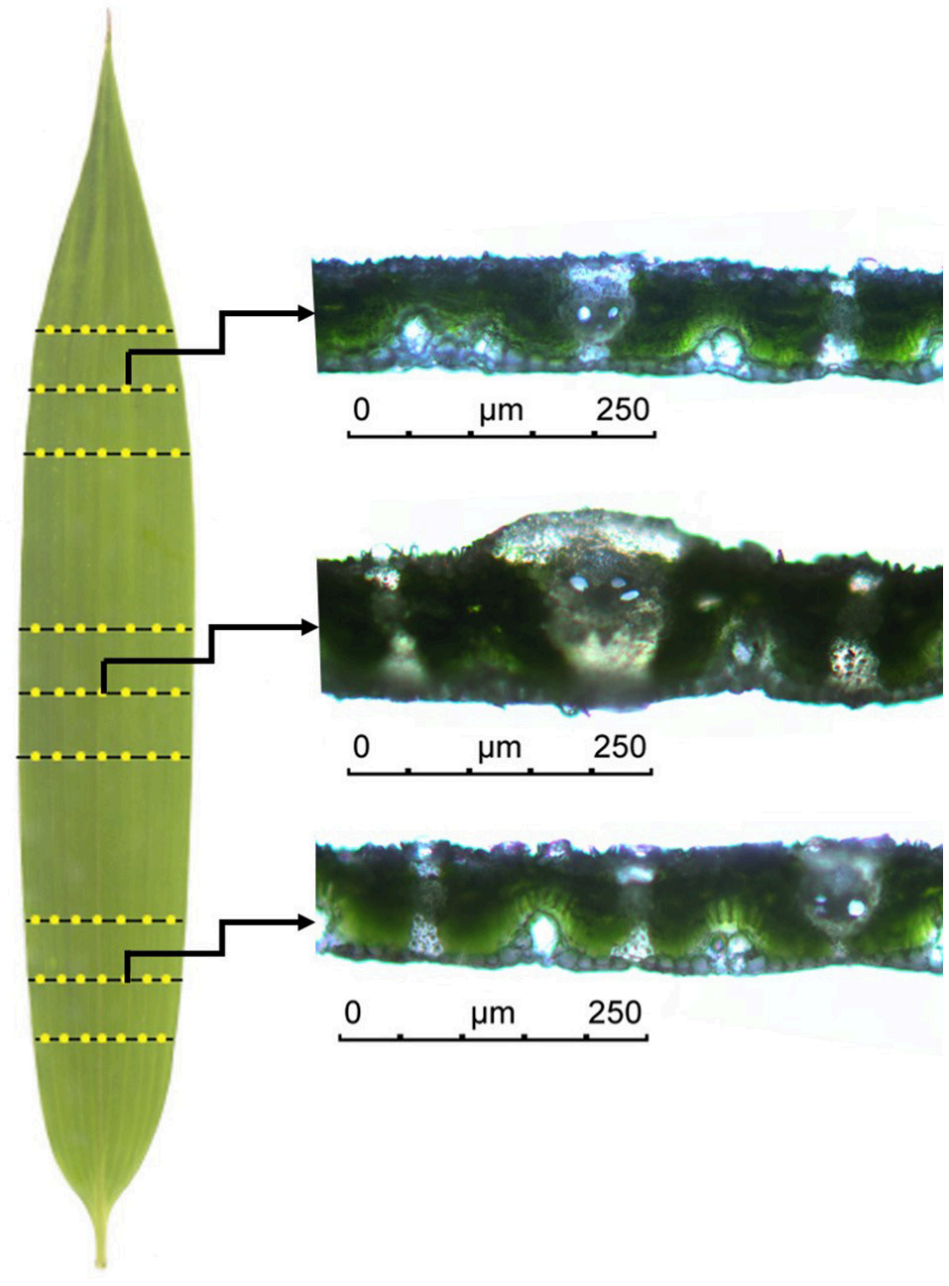
An illustration of how the mean thickness of a bamboo leaf is measured. The yellow points represent the locations for measuring the thickness. The data on the main vein were neglected because the values are extremely higher than those apart from the main vein.

In the aforementioned derivation, the key assumption is that leaf area is proportional to the 2-power of leaf thickness (i.e., Equation 5). In order to examine this hypothesis, we measured the leaf thickness of 100 leaves of *P. incarnata* randomly chosen from 210 leaves that had been used. Because the thickness at different locations on a leaf is different, the mean thicknesses was measured at sampled points that are not located on the main vein (Figure 2). Fresh leaves were punched early in the morning, and actual leaf thickness (LT) measured using hand sectioning. Leaf cross sections were photographed and measured under a light microscopy (DM 2500, a Leica Microsysterms CMS GmbH, Wetziar, Germany) at 20× magnification. We measured the distance between the adaxial and abaxial surfaces of the leaf at the base, middle and tip. Figure 2 illustrates the locations of sampled points in a leaf for measuring the mean thickness. Leaf areas were measured based on scanned images (Shi et al., 2015).

The scaling relationship was demonstrated using the reduced major axis (RMA; Milla and Reich, 2007). The reduced major axis is a linear-function fitting method. Different from the linear regression based on the ordinary least squares, the estimate of slope in the RMA is equal to the square root of the variance of response variables divided by the variance of independent variables (Hui et al., 2010). For the RMA and the ordinary least-squares (OLS), the applied target functions for minimizing the residuals to obtain the estimates of parameters are both residual sum of squares (RSS). The bootstrap percentile method (Efron and Tibshirani, 1993; Sandhu et al., 2011) was used to calculate the 95% confidence interval of the slope of the linear model. If a hypothesized power is not included within the 95% confidence interval of the slope (exponent) estimate, this will indicate an allometric relationship with the slope other than the tested value (in this case, 3/2). All analyses were done using R (version 3.2.2) (R Core Team, 2015).

The raw data used in the present study of leaf area, weight and thickness can be found in the Supplementary Material.

## Results

### Proportionality to leaf density and proportionality to leaf thickness

**Figure 3:**
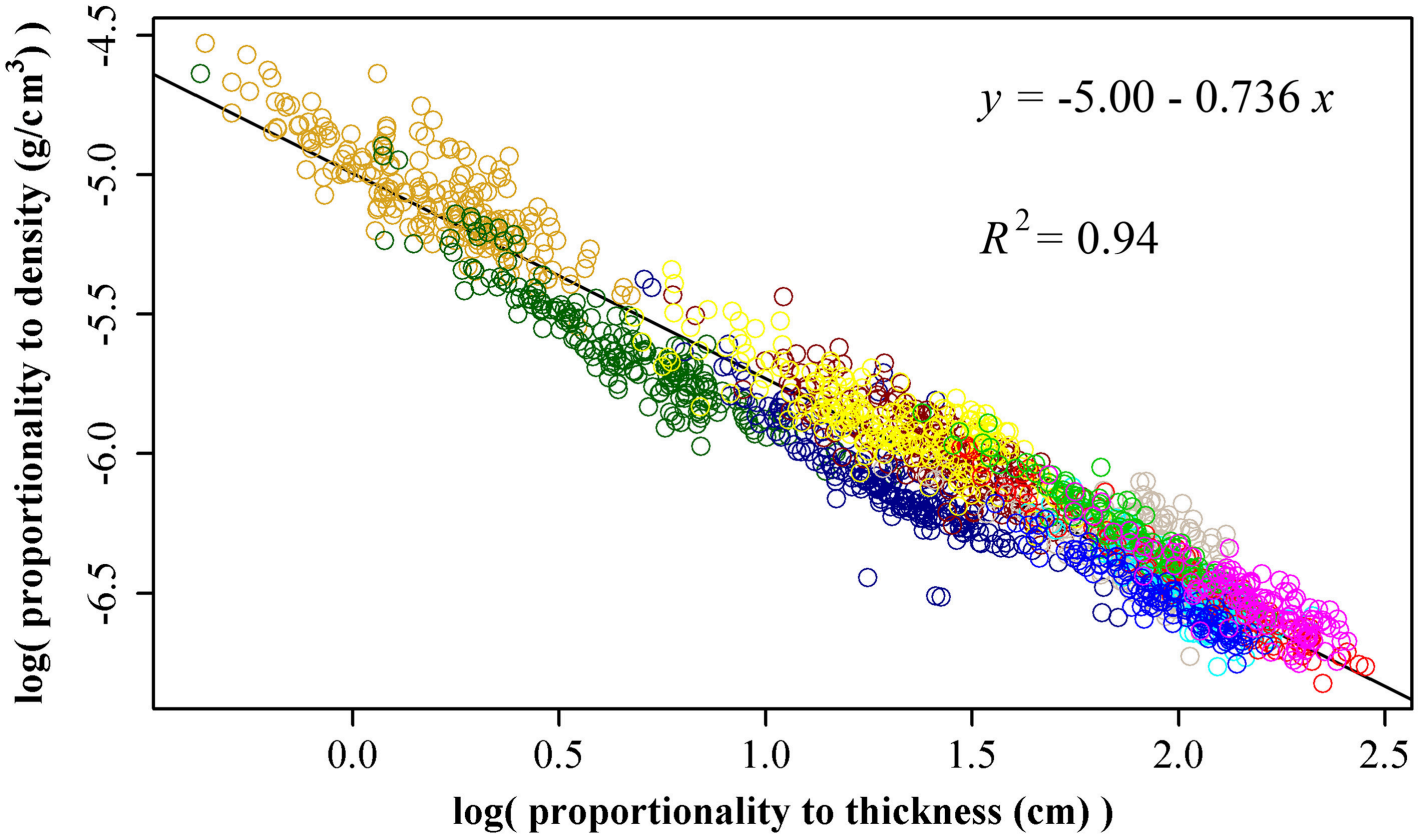
The linear fitting between the natural logarithm of the proportionality to leaf thickness and that of leaf density. Different colors represent different bamboo species, and there are totally 11 species. The proportionality of leaf thickness was obtained by *A*^1/2^, and the proportionality of leaf density was obtained by *W*·*A*^−3/2^.

Using 1864 data points, we obtained ln(ρ) = −5.00 −0.736 ln(*T*), RSS = 26.7, and *R*^2^ = 0.94 (Figure 3). That is, there is an inverse relationship between leaf thickness and leaf density. The 95% confidence interval of the slope is (−0.744, −0.727) based on 3000 bootstrap replications. Interestingly, the estimate of slope (i.e., *b* in Equation 9) is very approximate −3/4, which frequently appears in the investigation of the self-thinning law and metabolic theoretical ecology.

### Leaf mean thickness (T) and leaf surface area (A)

**Figure 4:**
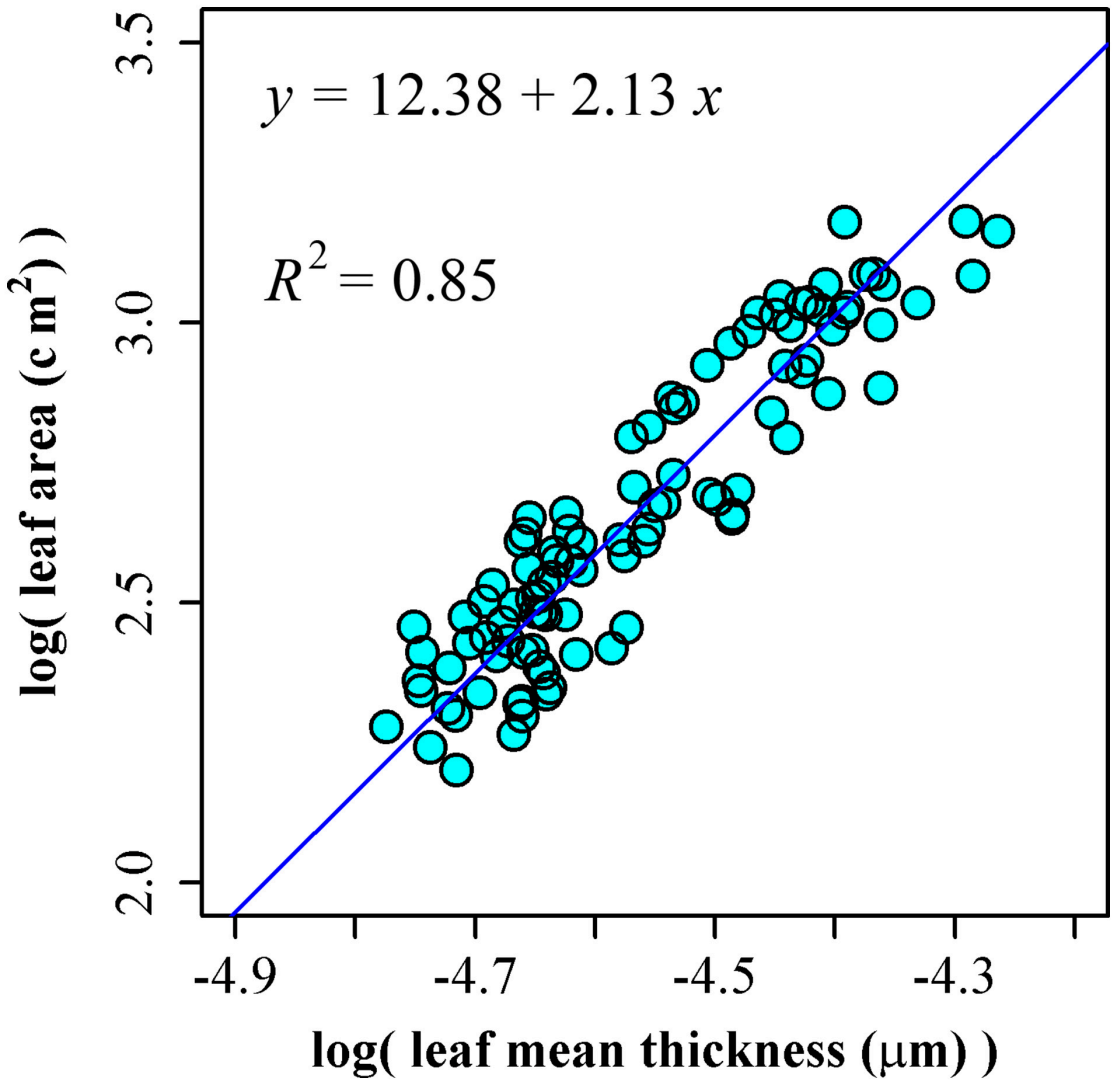
The linear fitting between the natural logarithm of leaf mean thickness and that of leaf area.

Using the leaf area and thickness data of 100 leaves of *P. incarnata*, we obtained ln(*A*) = 12.38 + 2.13 ln(*T*), RSS = 1.07, and *R*^2^ = 0.85 (Figure 4). The 95% confidence interval of slope is (1.98, 2.30) including the theoretical assumption of 2.

### Leaf surface area (A) and leaf fresh weight (W)

**Figure 5:**
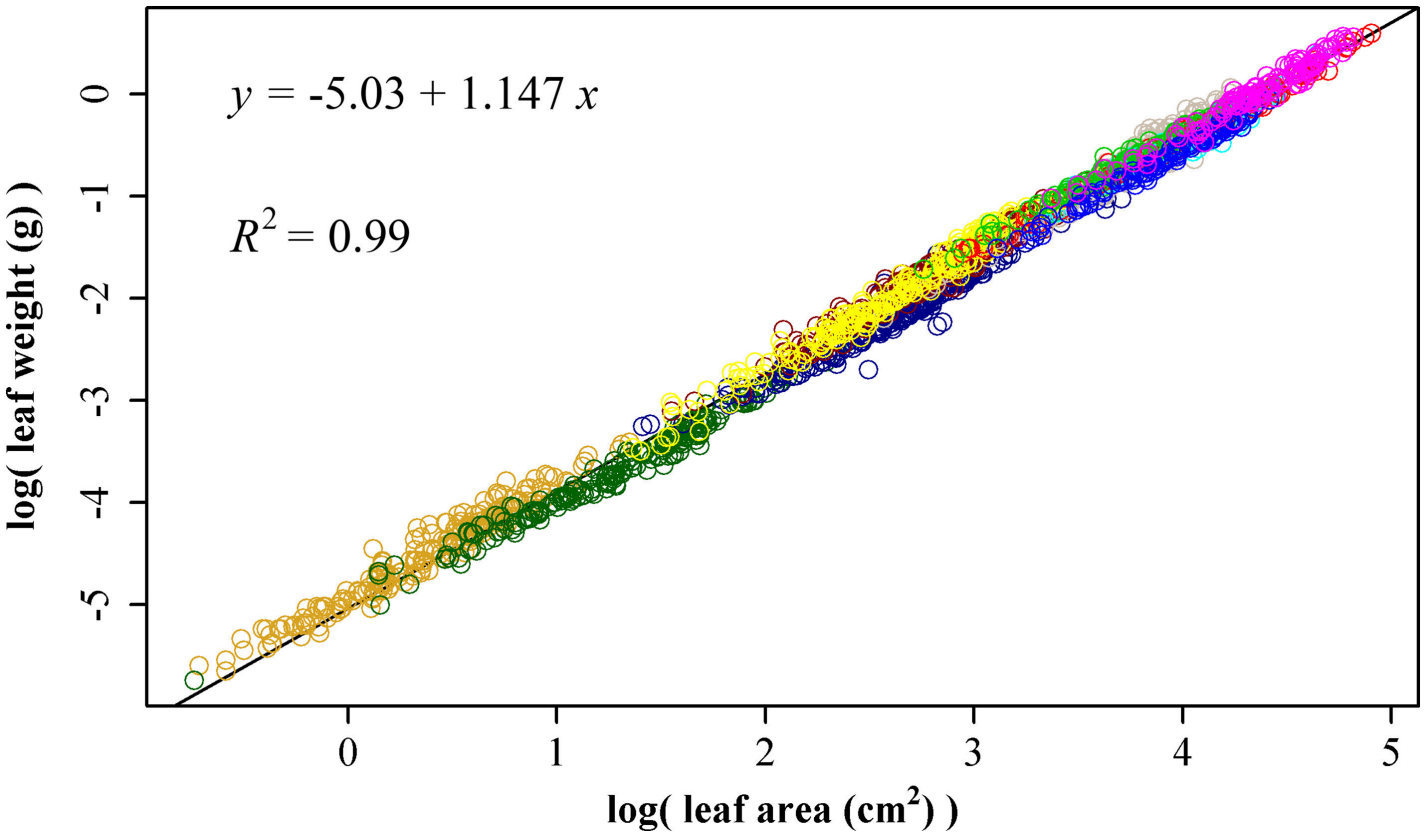
The linear fitting between the natural logarithm of leaf area and that of leaf fresh weight. Different colors represent different bamboo species, and there are totally 11 species.

Using 1864 data points, we obtained ln(*W*) = −5.03 + 1.147 ln(*A*), RSS = 26.4, and *R*^2^ = 0.99 (Figure 5). The 95% confidence interval of slope is (1.143, 1.152) based on 3000 bootstrap replications. The upper bound of the 95% confidence interval 1.152 is lower than 3/2, so the leaf weight-area allometry does not follow the 3/2-power law.

## Discussion

In this work, a negative relationship between leaf thickness and density was found in 11 bamboo species, consistent with the results for sclerophytes, mesophytes and succulents where the slope ranges from −0.46 to −0.82 (Vendramini et al., 2002). This negative correlation indicates that thinner leaves are also denser (Yano and Terashima, 2004), with more densely packed leaf cells (Pyankov et al., 1999; Vasfilov, 2012), allowing the thinner leaves to catch more light. Of course, leaf thickness and density can be affected by environmental factors, especially light. Leaf thickness tends to increase with decreasing rainfall, humidity, soil fertility and light (Beadle, 1966; Sobrado, 2008). Evergreen leaves are mostly thicker than deciduous leaves (Mooney and Rundel, 1979). Witkowski and Lamont (1991) also demonstrated that leaf area and thickness often vary independently with leaf position within a plant and between species. Here we showed that the correlation between leaf thickness and density was approximate −3/4, similar to that of the herbaceous plants (Vasfilov, 2012).

The possibility of the negative correlation between leaf thickness and density could stabilize LMA. Identifying the contributions of leaf thickness (*T*) and leaf density (ρ) to LMA is important in plant science. For example, Choong et al. (1992) found that the variation in LMA can be primarily driven by the variation of *T*. In contrast, Castro-Díez et al. (2000) confirmed that the LMA was correlated with ρ but not with *T* based on the observations of leaf inner structures of 52 European woody species. Xiong et al. (2016) also demonstrated that leaf density is the major cause of the variation in LMA across rice varieties. Villar et al. (2013) reported that ρ and *T* contributed equally to explaining the variation in LMA.

The correlation between leaf thickness and leaf area has been poorly studied. Niklas et al. (2007, 2009) found the leaf thickness increases with leaf area, but decreases for some species (e.g., *Auranticarpa rhombifolia* (A. Cunn. ex Hook.) L. W. Cayzer et al., *Ginkgo biloba* L., and *Populus nigra* L.). The estimate of slope between the log-transformed data of leaf area and mean leaf thickness in the present study is 2.13 based on 100 leaves of *P. incarnata*, supporting our assumption of a slope of 2 (95% confidence interval from 1.98 to 2.30).

**Figure 6:**
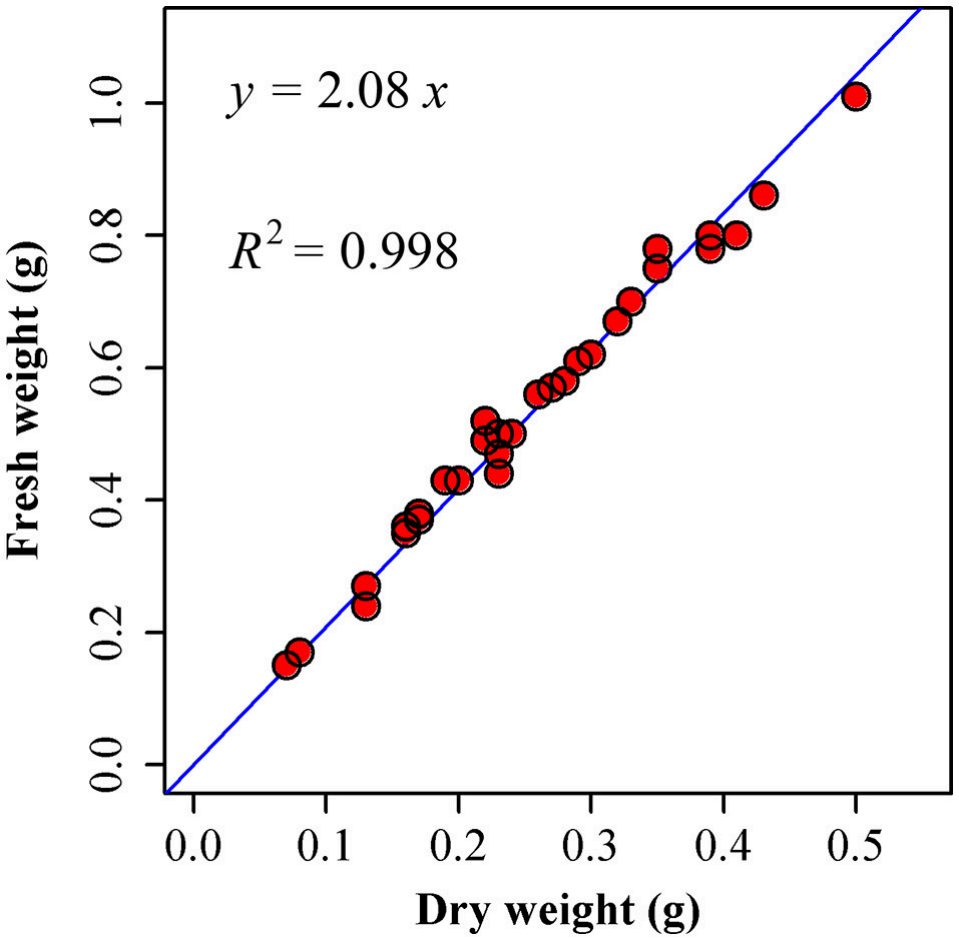
Comparison between the observed (points) and predicted fresh weights by using dry weights (the straight line) of *I. barbatus*. 30 leaves were randomly picked from these plants in the Nanjing Forestry University campus on 11 August, 2015. We measured their fresh weights as soon as we picked them from the plants (17:00 or so). Then these leaves were transferred to the oven at 60°C for 48 h. We measured their corresponding dry weights at 18:00 on 13 August, 2015. Then a linear regression was conducted between fresh weight (*W*_*F*_) and dry weight (*W*_*D*_). The estimate of slope is 2.0830 ± 0.0178, and *R*^2^ = 0.9979. That is, there is a strong proportional relationship between fresh and dry weights.

Because the scaling exponent governing the relationship between leaf dry and fresh weight across species is statistically indistinguishable from unity (Figure 6; Niklas et al., 2007; Shi et al., 2015), we directly used fresh weight (*W*_*F*_) rather than dry weight (*W*_*D*_):

(10) $$\begin{array}{r} W_{F}\propto W_{D}\propto A^{\left(b+3\right)/2}. \end{array}$$

Price and Enquist (2007) have proposed a model to account for the scaling of leaf area and weight based on the WBE model (West et al., 1999). The overall scaling exponent of leaf area to leaf weight was allometric (β = 1.08 in Milla et al., 2008; β = 1.10 in Milla and Reich, 2007; β = 1.02 in Niklas et al., 2007; β is from 0.859 to 1.299 in Pan et al., 2013). Here, we obtained ln (*W*) = −5.03 + 1.147 ln(*A*), RSS = 26.1, and *R*^2^ = 0.99 using 1864 data points. The 95% confidence interval of slope is (1.143, 1.152) based on 3000 bootstrap replications. Our scaling exponent 1.147 is in accordance with those reported in other studies (Niklas et al., 2007). Milla and Reich (2007) discovered the average exponent (1.10 with 95% CI 1.08–1.13) was significantly greater than 1 but lower than 3/2, and found that within each species large leaves deploy less surface area per unit dry mass than small ones. Niklas et al. (2007) also found that large leaves deploy less light-absorbing area per unit dry mass investment than small leaves in 943 species. Leaves are subject to strong selection pressure along gradients of aridity, solar radiation and nutrient availability that affect their size and shape (Price and Enquist, 2007). This leads to a wide range of variation in leaf area to leaf weight scaling exponent among species (Milla and Reich, 2007). The scaling relationship reflects the outcome of an evolutionary trade-off among ancestral metabolic, morphological and anatomical traits shared by all vascular plants (Niklas et al., 2007).

For bamboos, leaf area was demonstrated to be proportional to leaf mean thickness squared and also to be proportional to leaf length squared. Consequently, leaf volume can be expressed by leaf area to the power 3/2, which means that leaf volume, leaf area, leaf mean thickness (or leaf length) follow the similarity of principle of Thompson. The allometric relationship between leaf weight and leaf area based on the experimental data of 11 bamboo species was demonstrated to significantly deviate from the 3/2–power law. In other words, leaf weight is not proportional to leaf volume because leaf volume is the proportionality of leaf area to the power 3/2. To find the reason of the deviation to the 3/2–power law, we analyzed the relationship between the proportionality of leaf density and that of leaf mean thickness, and found a significant negative linear relationship between the log-transformed data of these two variables. That is, leaf density decreases with leaf size (represented by leaf mean thickness or leaf length or leaf area) increasing, which answers why leaf weight does not have a proportional relationship with leaf area to the power 3/2. The main conclusion in the present study that the negative scaling exponent of leaf thickness to density results in the deviation of the scaling exponent of leaf area to weight from the 3/2–power law could be potentially extended to other plants. However, the negative scaling exponent of leaf thickness to density (as demonstrated to be approximate to −3/4 for bamboos) might vary with different species of plants. In addition, whether the leaf area-thickness allometry follows the 2–power law for other plants also merits further investigation.

## Author contributions

SL and PS designed the experiment; LS, FL, YS, QW, and YD carried out this experiment; PS analyzed the data; PS, SL, CH, JG, and GR wrote the manuscript. All authors read and commented on this manuscript.

### Conflict of interest statement

The reviewer Z-XF declared a shared affiliation, with no collaboration, with one of the authors YS, to the handling Editor. The other authors declare that the research was conducted in the absence of any commercial or financial relationships that could be construed as a potential conflict of interest.

## Appendix

### The relationship between leaf surface area (*A*) and leaf length (*L*)

(A1) $$\begin{array}{r} A=\frac{1}{2}\int_{0}^{2\pi }{\left[r\left(\varphi \right)\right]}^{2}d\varphi \\ =\frac{1}{2}\int_{0}^{2\pi }{\left[l\cdot {\left(\left|\cos\frac{\varphi }{4}|+|\sin\frac{\varphi }{4}\right|\right)}^{-1/n}\right]}^{2}d\varphi \\ =\frac{l^{2}}{2}\left(\frac{2^{\left(3-\frac{1}{n}\right)}n\sqrt{\pi }\cdot Gamma\left[\frac{3}{2}-\frac{1}{n}\right]}{\left(n-2\right)\cdot Gamma\left[1-\frac{1}{n}\right]}-\frac{8n\cdot HypergeometricPFQ\left[\left\{\frac{1}{2},1\right\},\left\{\frac{3}{2}-\frac{1}{n}\right\},-1\right]}{n-2}\right). \end{array}$$

where “Gamma” represents the gamma function, and “HypergeometricPFQ” represents the generalized hypergeometric function. The detailed definitions for these two functions can be found in Wolfram MathWorld (http://mathworld.wolfram.com/). Because *n* is a constant, we have:

(A2) $$\begin{array}{r} A\propto l^{2}. \end{array}$$

Leaf length has demonstrated to be proportional to parameter *l* (Shi et al., 2015)

(A3) $$\begin{array}{r} L=\left(1+2^{-\frac{1}{n}}\right)\cdot l. \end{array}$$

Then we have:

$$\begin{array}{l} A\propto L^{2}. \end{array}$$
